# Impact of high-order time-delayed information on epidemic propagation in multiplex networks

**DOI:** 10.1016/j.idm.2025.08.007

**Published:** 2025-09-01

**Authors:** Zehui Zhang, Fang Wang, Lilin Liu, Lin Wang

**Affiliations:** a Laboratory of Intelligent Computing and Information Processing of the Ministry of Education and National Centre for Applied Mathematics in Hunan, Xiangtan University, Xiangtan, 411105, China; b The Third Xiangya Hospital of Central South University, Changsha, 410013, China; c Department of Mathematics and Statistics, University of New Brunswick, Fredericton, NB, E3B 5A3, Canada

**Keywords:** Information-disease coupled dynamics, High-order delayed, Multiplex network, Microscopic Markov chain

## Abstract

Traditional epidemic models often overlook disease incubation periods and high-order social interactions, limiting their ability to capture real-world transmission dynamics. To address these gaps, we develop a stochastic model that integrates both factors, investigating their combined effects on information diffusion and disease spread. Our framework consists of a two-layer network: an awareness layer, where disease-related information propagates through high-order delayed interactions, and an epidemic layer, where disease transmission follows an SIS model with incubation delays. Using a Markov chain approach, we derive outbreak thresholds and perform numerical simulations to assess the impact of delayed awareness adoption on epidemic outcomes. High-order delayed interactions accelerate information spread compared to traditional pairwise models. Interestingly, while incubation periods increase the risk of hidden transmission, they also provide a crucial window for awareness diffusion, potentially mitigating outbreaks. This dual role of incubation prolonging undetected transmission while enabling proactive awareness dissemination underscores the importance of synchronizing public health interventions with disease incubation phases.

## 1 Introduction

Throughout history, epidemics have posed significant threats to human societies, continuously endangering global health and causing widespread fatalities (Morens et al., 2004, 2008; Parrish et al., 2008). Notable examples include Smallpox (Gani et al., 2001), SARS (Peiris et al., 2004), and COVID-19 (Oveson et al., 2025). Understanding the dynamics of epidemic transmission within communities—particularly in relation to outbreak thresholds and transmission scales—is crucial for developing effective prevention and control strategies. The rapid advancement of complex network science has enabled researchers to employ network-based approaches to explore the mechanisms of epidemic spread (Shi et al., 2025; Alrawas et al., 2024; Azizi et al., 2020).

With the rise of digital communication, awareness of epidemics spreads rapidly following an outbreak, primarily through social media platforms such as Facebook, WeChat, and Microblog (Li et al., 2020; Gao et al., 2024; Xia et al., 2019). Access to timely information allows individuals to adopt protective measures—such as wearing masks, getting vaccinated, and working from home—to reduce their risk of infection (Fan et al., 2016; Wang et al., 2020; Cheng et al., 2022). These behavioral responses can significantly influence epidemic dynamics, underscoring the importance of understanding the interplay between information dissemination and disease transmission.

Most existing models that couple epidemic and information propagation rely on multiplex network frameworks (Yang et al., 2019; Zhang et al., 2025b; Xie & Huo, 2024; Feng et al., 2023). Granell et al. (Granell et al., 2013) demonstrated that awareness dynamics on multiplex networks can influence epidemic thresholds and reduce disease incidence through interactions between virtual and physical networks. Similarly, You et al. (You et al., 2023) proposed a coupled information-disease model showing that positive emotions suppress outbreaks, whereas negative emotions amplify transmission. Sun et al. (Sun et al., 2024) introduced an SIRS-UAU model on multiplex networks, revealing that mass media influences epidemic thresholds and prevalence, with awareness leading to varying levels of self-protection. Additionally, Feng et al. (Feng et al., 2024a) developed a time-varying multiplex network model incorporating community structure, highlighting its role in shaping the interplay between information diffusion and epidemic spread.

However, many of these studies assume that individuals within a network share uniform characteristics. In reality, individuals exhibit heterogeneity, leading to diverse behavioral responses. Several models have been proposed to account for this variability. For instance, Feng et al. (Feng et al., 2024b) developed a two-layer signed multiplex network model, demonstrating that individual heterogeneity and positive social relationships enhance awareness diffusion while mitigating disease spread. Xu et al. (Xu et al., 2024) showed that awareness heterogeneity significantly reduces epidemic transmission, while Pan and Yan (Pan & Yan, 2018) highlighted how individual differences impact epidemic thresholds and spreading dynamics. Scatà et al. (Scatà et al., 2016) examined the coevolution of epidemic and awareness spreading, emphasizing the role of heterogeneity and preventive isolation in delaying outbreaks and improving resilience. Wu et al. (Wu et al., 2022) further explored how variations in information literacy influence epidemic diffusion, revealing that higher literacy levels promote awareness adoption and suppress disease transmission.

Despite these advancements, most two-layer models primarily focus on pairwise interactions between individuals, overlooking the higher-order structures and collective behaviors inherent in real-world social networks. This simplification limits our ability to fully capture the complexity of information dissemination and epidemic spread. Recognizing this gap, researchers have begun incorporating high-order structures into various types of complex networks, including online social networks (Alvarez-Rodriguez et al., 2021; Gambuzza et al., 2021), neural networks (Wu et al., 2023; Li et al., 2024), and ecological networks (Raj et al., 2024; Silk et al., 2022). Iacopini et al. (Iacopini et al., 2019) introduced a high-order social contagion model using simplicial complexes, demonstrating that group interactions can induce discontinuous transitions and bistable regions in complex systems. Zhang et al. (Zhang et al., 2024) incorporated herd-awareness into a two-layer metapopulation model, showing that self-herd awareness effectively curbs epidemic spread. Ye et al. (Ye et al., 2025) developed a reaction-diffusion rumor propagation model using simplicial complexes on multiplex networks, highlighting how high-order interactions influence spatial patterns and diffusion dynamics. Additionally, You et al. (You et al., 2024) introduced a coupled information-disease model on temporal simplicial networks, revealing that message fatigue can lead to a two-stage shift in spreading dynamics, where excessive information dissemination may backfire.

While existing research has extensively examined information dissemination mechanisms, the role of incubation periods and time-delay effects in epidemic spreading remains largely unexplored (Wang et al., 2017, 2018; Zhang et al., 2022). The incubation period is a crucial aspect of disease transmission, as every infected individual undergoes a latency phase before becoming infectious. These incubation durations vary significantly—from hours to days—and can involve asymptomatic carriers who unknowingly spread the disease. Individuals in the incubation phase who engage in social activities may serve as hidden transmitters, complicating containment efforts.

In our recent work (Zhang et al., 2025a), we introduced a time-delay framework to model individual disease incubation within the context of information-disease interactions on multiplex networks. This approach better aligns awareness dynamics with real-world epidemic scenarios. However, the model did not account for high-order neighbor interactions. Building on that foundation, our current study explores the impact of time-delay effects in high-order information dissemination on disease transmission. We propose a coupled information-disease spreading model that incorporates high-order delayed effects, called information-disease coupling propagation model (IDCPM) with high-order delayed, providing a more comprehensive understanding of the interplay between information diffusion and epidemic dynamics. Through extensive numerical simulations, our analytical results demonstrate that accounting for incubation periods enhances the accuracy of epidemic transmission modeling, offering valuable insights for epidemic prevention and control strategies.

The remainder of this paper is structured as follows. In Section 2, we propose an information-disease coupled spreading model that incorporates disease incubation periods and high-order relationships within a multiplex network, and proceed to conduct a detailed model analysis. In Section 3, we perform extensive numerical simulations to verify our theoretical conclusions. Section 4 offers an in-depth discussion comparing the contributions of pairwise and high-order interaction patterns to information dissemination. Finally, Section 5 summarizes the key findings and discusses the potential limitations of our model.

## 2 IDCPM with high-order delayed interactions

The most foundational propagation model was proposed by Granell (Granell et al., 2013), marking the first description of coupled information and disease spread on multiplex networks. This model assumes propagation occurs exclusively through pairwise interactions. However, Iacopini et al. (Iacopini et al., 2019) later demonstrated that pairwise interaction models fail to fully capture real-world infection dynamics. To address this limitation, the concept of group interactions was introduced, incorporating simplicial complexes to represent high-order structures within the network. A *k*-simplex, denoted as *M*, is defined as a pair (*V*, *E*), where *V* is a set of *N* nodes with |*V*| = *N*, and *E* represents the set of simplicial complexes contained within *M*. The order of *M*, denoted as *d*, corresponds to the dimension of the simplex. Specifically, a *d*-simplex is a convex polyhedron with *d* + 1 vertices. For example, a 0-simplex consists of a single vertex, a 1-simplex represents an edge connecting two vertices, a 2-simplex forms a triangle with three vertices, and so forth.

The complexity of disease transmission arises from individual variations in incubation periods, leading to delays in information spread. Here, we explore the impact of these incubation delays in detail. Due to physiological differences, the incubation period of an individual varies. For simplicity, we assume the incubation period is measured in days and denote it as *τ*_*i*_, rounded to the nearest integer. We assume that each individual is in one of four states: *US* (unaware-susceptible), *AS* (aware-susceptible), *UI* (unaware-infected), and *AI* (aware-infected). Accordingly, we identify three cases:

Case I: If the incubation period of node *i* is at most one day (*τ*_*i*_ ≤ 1), the node is assumed to be immediately exposed in the next time step, bypassing the *UI*-state and transitioning directly to the *AI*-state. In this case, the system consists of only three states: *US*, *AS*, and *AI*.
Case II: If node *i* is still within its incubation period (1 < *t* < *τ*_*i*_), an unaware infected node remains in the *UI*-state. Under these circumstances, the system includes four states: *US*, *UI*, *AS*, and *AI*.
Case III: When the incubation period ends (1 < *τ*_*i*_ ≤ *t*), the node in the *UI*-state immediately develops symptoms and transitions to the *AI*-state. At this stage, all four states (*US*, *UI*, *AS*, and *AI*) coexist in the system.

**Fig. 1:**
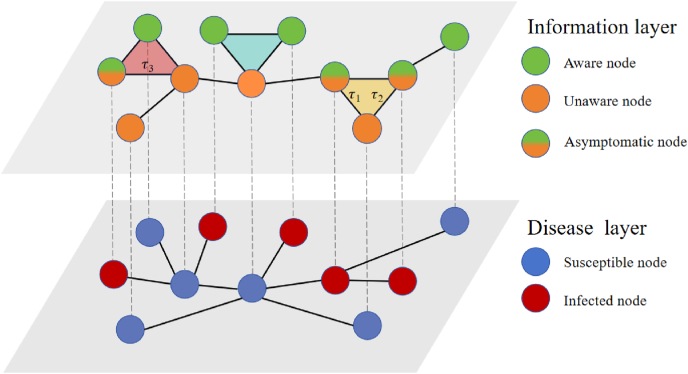
Diagram of the information-disease coupled propagation model with high-order delays on multiplex networks. The upper layer represents the information layer, consisting of nodes in the *A*-state and *U*-state. The lower layer represents the disease layer, comprising nodes in the *I*-state and *S*-state.

To accurately characterize the coupled dynamics of information diffusion and disease transmission, we introduce a high-order delayed model set within a two-layer multiplex network, as illustrated in Fig. 1.

Remark 1 In the upper layer of the network, various mechanisms governing the transition of unaware nodes to an informed state are illustrated. The blue triangle represents regions where information propagates within the 2-simplex. The yellow triangle denotes areas where information spreads after a delay of max(*τ*_1_, *τ*_2_). The red triangle indicates regions where information propagates after a time interval of *τ*_3_. Notably, aware asymptomatic nodes in the upper layer always correspond to infected nodes in the lower layer.

The network comprises two layers: the upper layer (information layer) and the lower layer (disease layer). The upper layer characterizes the spread of awareness among individuals, incorporating group interactions modeled through 2-simplex structures, and the lower layer captures the transmission dynamics, accounting for both symptomatic and asymptomatic infections.

This model is based on two key assumptions (Fan et al., 2022): one is both network layers are unweighted and undirected; the other is although nodes in both layers correspond one-to-one, their topological structures differ.

In the upper layer, information diffusion follows a *U*-*A*-*U* (unaware-aware-unaware) process, where each individual exists in one of two states: the *U*-state (unaware) or the *A*-state (aware). Unaware individuals can acquire disease-related information through two mechanisms. (1) Pairwise interactions (1-simplex transmission): An unaware node becomes aware with probability *λ* when interacting with an aware neighbor, and (2) Group interactions (2-simplex transmission): Within a 2-simplex, two aware nodes jointly spread information to an unaware node with probability *λ*_△_. Additionally, aware nodes may forget disease-related information and revert to the unaware state with probability *δ*.

In the lower layer, the *S*–*I*–*S* (susceptible–infected–susceptible) model is used to describe epidemic spread. Each node can be in one of two states: susceptible (*S*) or infected (*I*). At each discrete time step, a susceptible node may become infected upon contact with an infected neighbor, with probability *β*. Simultaneously, infected nodes recover and return to the susceptible state with probability *μ*.

Let *β*^*U*^ and *β*^*A*^ denote the infection rates for unaware and aware individuals, respectively. Typically, individuals who are aware of an epidemic adopt protective behaviors that reduce their risk of infection. We thus assume a reduced infection rate for aware nodes, given by *β*^*A*^ = *γβ*^*U*^, where *γ*(0 ≤ *γ* ≤ 1) is the disease-transmission reduction factor. In particular, *γ* = 0 corresponds to full immunity for aware individuals, i.e., *β*^*A*^ = 0.

**Fig. 2:**
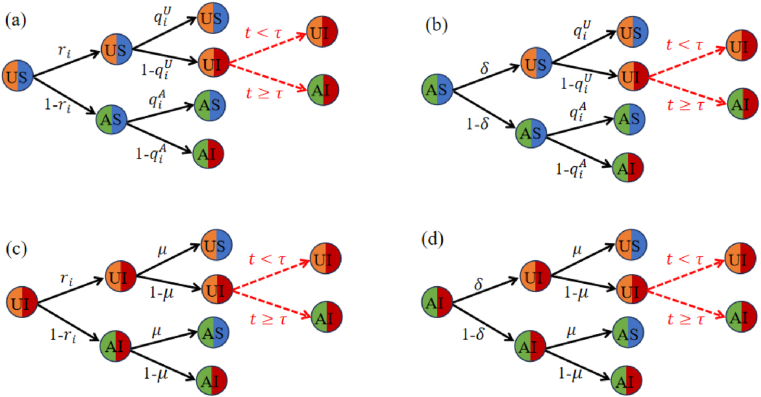
Transition probability tree for node states. Each node can be in one of four states: *US*, *UI*, *AS*, or *AI*.

Fig. 2 illustrates the transition probability trees for node states across three cases. Each node can be in one of four states: *US* (unaware and susceptible), *UI* (unaware and infected), *AS* (aware and susceptible), and *AI* (aware and infected).

Remark 2 Within the system, each node resides in one of four states: *US*, *AS*, *UI*, or *AI*. In Figure 2*,* black arrows sequentially represent the processes of information propagation and disease transmission. Notably, red arrows specifically indicate transitions associated with nodes in the *UI* state. When *t* < *τ*, these arrows signify that asymptomatic nodes have not yet developed symptoms. Conversely, when *t* ≥ *τ*, they indicate that the incubation period has ended, and the node has transitioned to the *AI* state.

Typically, the incubation period follows a long-tailed distribution. Based on our previous findings (Zhang et al., 2025a), among several candidate distributions—such as exponential, Weibull, and Gamma distribution, particularly, the lognormal distribution exhibits the most significant influence on the model. In fact, the lognormal incubation period function characterizes the probability distribution of the time interval between infection and the onset of symptoms. It reflects that most individuals have an incubation period clustered around a typical value, while interindividual variability leads to a fraction exhibiting shorter or longer periods, resulting in a right-skewed (long-tailed) distribution. This form is commonly used to fit empirical epidemiological data, as incubation periods are often influenced by multiple biological and environmental factors in a multiplicative manner, a process that naturally gives rise to a lognormal distribution. Consequently, we assume that the incubation period for each individual, denoted by T, follows an independent and identically distributed lognormal distribution:

(1) $$T∼f\left(\tau \right)=\frac{1}{\tau \sigma \sqrt{2\pi }}\exp\left(-\frac{{\left(\ln\;\tau -\theta \right)}^{2}}{2\sigma^{2}}\right),\;\;\text{for}\;\tau \geq 0,$$

where *θ* and *σ* represent the mean and standard deviation of the lognormal distribution, respectively.

In what follows, we employ the microscopic Markov chain approach (MMCA) method to initially characterize the proposed high-order delayed information-disease coupled propagation and then derive the epidemic threshold *β*_*c*_. In this model, each individual can be in one of four states: *US* (unaware-susceptible), *AS* (aware-susceptible), *UI* (unaware-infected), and *AI* (aware-infected). The corresponding probabilities for node *i* at time step *t* are denoted as $p_{i}^{US}\left(t\right)$, $p_{i}^{AS}\left(t\right)$, $p_{i}^{UI}\left(t\right)$, and $p_{i}^{AI}\left(t\right)$, respectively. These probabilities satisfy the normalization condition:

$$p_{i}^{US}\left(t\right)+p_{i}^{AS}\left(t\right)+p_{i}^{UI}\left(t\right)+p_{i}^{AI}\left(t\right)=1.$$

In the upper network (information layer), the probability *r*_*i*_(*t*) that an unaware node *i* remains uninformed at time *t* is the product of two factors:

$$r_{i}\left(t\right)=r_{i}^{↔}\left(t\right)r_{i}^{△}\left(t\right),$$

where $r_{i}^{↔}\left(t\right)$ is the probability that node *i* is not informed by its aware neighbors via 1-simplex interactions and $r_{i}^{△}\left(t\right)$ is the probability that node *i* is not informed by its aware neighbors via 2-simplex interactions. These probabilities are given by:

(2) $$\left\{\begin{array}{ll} & r_{i}^{↔}\left(t\right)=\underset{j}{\prod }\left[1-a_{ij}p_{j}^{A}\left(t\right)\lambda \right],\\ & r_{i}^{△}\left(t\right)=\underset{j,k}{\prod }\left[1-a_{\mathrm{ijk}}p_{j}^{A}\left(t\right)p_{k}^{A}\left(t\right)\lambda_{△}\right], \end{array}\right)$$

where $A={\left(a_{ij}\right)}_{N\times N}$ represents the adjacency matrix of the upper network, indicating whether nodes form a 1-simplex (*a*_*ij*_ = 1 if so, otherwise 0). Similarly, for a 2-simplex, *a*_*ijk*_ = 1 if nodes *i*, *j*, *k* form a triangle; otherwise, *a*_*ijk*_ = 0.

In the lower network (disease layer), the probabilities that unaware and aware nodes are not infected at time *t* are:

(3) $$\left\{\begin{array}{l} q_{i}^{U}\left(t\right)=\underset{j}{\prod }\left[1-b_{ij}p_{j}^{I}\left(t\right)\beta^{U}\right],\\ q_{i}^{A}\left(t\right)=\underset{j}{\prod }\left[1-b_{ij}p_{j}^{I}\left(t\right)\beta^{A}\right], \end{array}\right)$$

where $B={\left(b_{ij}\right)}_{N\times N}$ is the adjacency matrix of the disease layer: *b*_*ij*_ = 1 if an edge exists between *i* and *j*, and *b*_*ij*_ = 0 if there is no edge between node *i* and node *j*.

According to Fig. 2, we derive the transition probabilities for three cases by using Eqs. (2), (3).

**Case I:** *τ*_*i*_ ≤ 1. The transition probabilities at node *i* for transitions among the three states *US*, *AS*, and *AI* from *t* to *t* + 1 are governed as follows:

(4) $$\left\{\begin{array}{ll} p_{i}^{US}\left(t+1\right) & =p_{i}^{US}\left(t\right)r_{i}\left(t\right)q_{i}^{U}\left(t\right)+p_{i}^{AS}\left(t\right)\delta q_{i}^{U}\left(t\right)+p_{i}^{AI}\left(t\right)\delta \mu ;\\ p_{i}^{AS}\left(t+1\right) & =p_{i}^{US}\left(t\right)\left[1-r_{i}\left(t\right)\right]q_{i}^{A}\left(t\right)+p_{i}^{AS}\left(t\right)\left(1-\delta \right)q_{i}^{A}\left(t\right)\\ & +p_{i}^{AI}\left(t\right)\left(1-\delta \right)\mu ;\\ p_{i}^{AI}\left(t+1\right) & =p_{i}^{US}\left(t\right)r_{i}\left(t\right)\left[1-q_{i}^{U}\left(t\right)\right]+p_{i}^{US}\left(t\right)\left[1-r_{i}\left(t\right)\right]\left[1-q_{i}^{A}\left(t\right)\right]\\ & +p_{i}^{AS}\left(t\right)\left[\delta \left(1-q_{i}^{U}\left(t\right)\right)+\left(1-\delta \right)\left(1-q_{i}^{A}\left(t\right)\right)\right]\\ & +p_{i}^{AI}\left(t\right)\left(1-\mu \right). \end{array}\right)$$

**Case II:** 1 < *t* < *τ*_*i*_. The transition probabilities are:

(5) $$\left\{\begin{array}{ll} p_{i}^{US}\left(t+1\right) & =p_{i}^{US}\left(t\right)r_{i}\left(t\right)q_{i}^{U}\left(t\right)+p_{i}^{AS}\left(t\right)\delta q_{i}^{U}\left(t\right)+p_{i}^{AI}\left(t\right)\delta \mu +p_{i}^{UI}\left(t\right)r_{i}\left(t\right)\mu ;\\ p_{i}^{AS}\left(t+1\right) & =p_{i}^{US}\left(t\right)\left[1-r_{i}\left(t\right)\right]q_{i}^{A}\left(t\right)+p_{i}^{AS}\left(t\right)\left(1-\delta \right)q_{i}^{A}\left(t\right)\\ & +p_{i}^{AI}\left(t\right)\left(1-\delta \right)\mu +p_{i}^{UI}\left(t\right)\left[1-r_{i}\left(t\right)\right]\mu ;\\ p_{i}^{UI}\left(t+1\right) & =p_{i}^{US}\left(t\right)r_{i}\left(t\right)\left[1-q_{i}^{U}\left(t\right)\right]+p_{i}^{AS}\left(t\right)\delta \left[1-q_{i}^{U}\left(t\right)\right]\\ & +p_{i}^{UI}\left(t\right)r_{i}\left(t\right)\left(1-\mu \right)+p_{i}^{AI}\left(t\right)\delta \left(1-\mu \right);\\ p_{i}^{AI}\left(t+1\right) & =p_{i}^{US}\left(t\right)\left[1-r_{i}\left(t\right)\right]\left[1-q_{i}^{A}\left(t\right)\right]+p_{i}^{AS}\left(t\right)\left(1-\delta \right)\left[1-q_{i}^{A}\left(t\right)\right]\\ & +p_{i}^{UI}\left(t\right)\left[1-r_{i}\left(t\right)\right]\left(1-\mu \right)+p_{i}^{AI}\left(t\right)\left(1-\delta \right)\left(1-\mu \right). \end{array}\right)$$

It is important to note the presence of the *UI*-state in this case, as it represents infected individuals in the incubation period who are not yet aware of their infection (see the red arrow labeled *t* < *τ* in Fig. 2).

**Case III:** 1 < *τ*_*i*_ ≤ *t*.

The transition probabilities of node *i* in the *UI* and *AI* states differ from those in Eq. (5), and are given by:

(6) $$\left\{\begin{array}{ll} p_{i}^{UI}\left(t+1\right) & =p_{i}^{US}\left(t\right)r_{i}\left(t\right)\left[1-q_{i}^{U}\left(t\right)\right]+p_{i}^{AS}\left(t\right)\delta \left[1-q_{i}^{U}\left(t\right)\right]\\ & +p_{i}^{AI}\left(t\right)\delta \left(1-\mu \right)+p_{i}^{UI}\left(t\right)r_{i}\left(t\right)\left(1-\mu \right)\\ & -p_{i}^{UI}\left(t-\tau_{i}+1\right){\left[r_{i}\left(t\right)\left(1-\mu \right)\right]}^{\tau_{i}};\\ p_{i}^{AI}\left(t+1\right) & =p_{i}^{US}\left(t\right)\left[1-r_{i}\left(t\right)\right]\left[1-q_{i}^{A}\left(t\right)\right]+p_{i}^{AS}\left(t\right)\left(1-\delta \right)\left[1-q_{i}^{A}\left(t\right)\right]\\ & +p_{i}^{UI}\left(t\right)\left[1-r_{i}\left(t\right)\right]\left(1-\mu \right)+p_{i}^{AI}\left(t\right)\left(1-\delta \right)\left(1-\mu \right)\\ & +p_{i}^{UI}\left(t-\tau_{i}+1\right){\left[r_{i}\left(t\right)\left(1-\mu \right)\right]}^{\tau_{i}}. \end{array}\right)$$

**Fig. 3:**
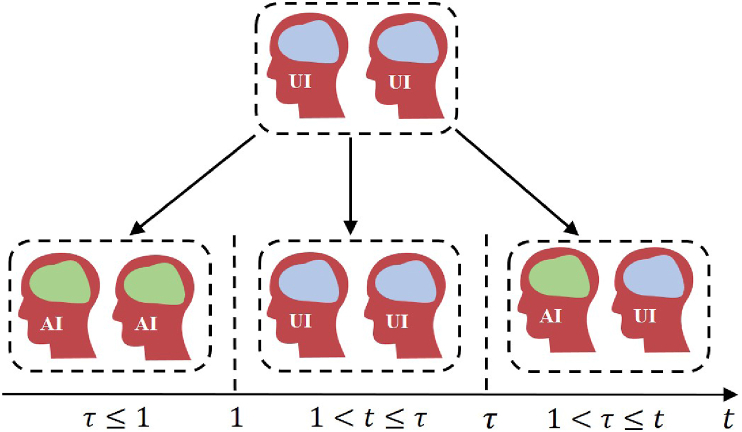
Schematic diagram of *UI*-state's transition in individuals with incubation periods under three cases. When *τ* < 1, the *UI*-state transitions to the *AI*-state immediately; when 1 < *t* ≤ *τ*, the *UI*-state persists as the current state at time *t* + 1, as the individual remains in the incubation period; once *t* > *τ*, a portion of individuals in *UI*-state will transition to the *AI*-state.

The difference among there three cases mainly focus on the calculation of $P_{i}^{UI}$. To better understand this difference, we present a schematic diagram of transition of *UI*-state in Fig. 3.

The term *τ*_*i*_ in Eq. (6) signifies the number of information and disease propagation cycles that a node in the *UI* state undergoes before it becomes exposed and transitions into the *AI* state.

As *t* → *∞*, the propagation of information and the spread of disease in our model are expected to reach a stable steady state. Accordingly, the steady-state equations for the four possible node states can be expressed as $p_{i}^{⋄}\left(t+1\right)=p_{i}^{⋄}\left(t\right)=p_{i}^{⋄}$, where ⋄ ∈ {*US*, *AS*, *UI*, *AI*}. When the transmission rate *β* approaches the epidemic threshold, the fraction of infected individuals tends to zero. Thus, the proportion of infected nodes can be approximated by $\epsilon_{i}=p_{i}^{I}=p_{i}^{UI}+p_{i}^{AI}\ll 1$ and hence, Eq. (3) can be approximated by

$$q_{i}^{U}\approx 1-\beta^{U}\underset{j}{\sum }b_{ij}\epsilon_{j},\;q_{i}^{A}\approx 1-\beta^{A}\underset{j}{\sum }b_{ij}\epsilon_{j}.$$

Neglecting higher-order terms, Eq. (4) ∼(6) can be simplified as

(7) $$\left\{\begin{array}{ll} & p_{i}^{U}=p_{i}^{U}r_{i}+p_{i}^{A}\delta ,\\ & p_{i}^{A}=p_{i}^{U}\left(1-r_{i}\right)+p_{i}^{A}\left(1-\delta \right),\\ & \mu \epsilon_{i}=\left(p_{i}^{A}\beta^{A}+p_{i}^{U}\beta^{U}\right)\underset{j}{\sum }b_{ij}\epsilon_{j}. \end{array}\right)$$

Since $p_{i}^{I}$ is very small, we can approximate $p_{i}^{A}=p_{i}^{AS}+p_{i}^{AI}\approx p_{i}^{AS}$, and $p_{i}^{U}=p_{i}^{US}+p_{i}^{UI}\approx 1-p_{i}^{A}$. With these approximations, the last equation in Eq. (7) further simplifies to

(8) $$\underset{j}{\sum }\left\{\left[1-\left(1-\gamma \right)p_{i}^{A}\right]b_{ji}-\frac{\mu }{\beta }\xi_{ji}\right\}\epsilon_{j}=0,$$

where *ξ*_*ji*_ = 1 if *j* = *i* and *ξ*_*ji*_ = 0 if *j* ≠ *i*.

Let $h_{ij}=\left[1-\left(1-\gamma \right)p_{i}^{A}\right]b_{ji}$ be the (i,j)-th element of matrix *H*, and let Λ_max_(*H*) denote its largest eigenvalue. Then Eq. (8) can be interpreted as an eigenvalue problem of matrix *H*, and the epidemic threshold is given by

(9) $$\beta_{c}=\frac{\mu }{\Lambda_{\max}\left(H\right)}.$$

This result highlights that *β*_*c*_ is independent of the incubation period *τ*_*i*_ and is primarily influenced by the probability of being in the *A*-state.

## 3 Numerical simulations

In this section, we explore the performance of the proposed time-delayed high-order information-disease coupled propagation model within a two-layer multiplex network. To achieve this, we first construct the multiplex network.

The upper-layer network is generated using the random simplicial complex (RSC) model. To generate a *d*-dimensional simplex, *d* + 1 parameters are required: the network size *N* and *d* probabilities {*p*_1_, *p*_2_, *…*, *p*_*d*_}, where each probability corresponds to the formation of a simplex of the respective dimension. In this study, we focus on the case of *d* = 2, requiring three parameters {*N*, *p*_1_, *p*_2_}. The process for generating a simplicial complex with *d* = 2 is as follows:

• First, randomly select two nodes (*i*, *j*) from the *N* nodes and create an edge (i.e., a 1-simplex) with probability *p*_1_.
• Next, randomly select three nodes (*i*, *j*, *l*) from the *N* nodes and form a 2-simplex with probability *p*_2_.

As a result, the average degree of the network is given by

(10) $$\langle k\rangle =\left(N-1\right)p_{1}+2\langle k_{△}\rangle \left(1-p_{1}\right),$$

where ⟨*k*_△_⟩ = (*N* − 1)(*N* − 2)*p*_2_/2 is the average degree of the 2-simplex (Iacopini et al., 2019).

The lower-layer network is generated using an Erdős-Rényi (ER) model with a connection probability *p*_3_. In our study, we set *p*_1_ = 0.006, *p*_2_ = 0.0004, and *p*_3_ = 0.006.

In our simulations, each layer of the network comprises *N* = 1000 nodes. Initially, 1 % of the nodes are randomly selected as infected. The pairwise and 2-simplex information propagation rates, denoted as *λ* and *λ*_△_, the information forgetting rate *δ*, the disease recovery rate *μ*, and the disease transmission attenuation factor *γ* are set to 0.15, 0.15, 0.5, 0.4, and 0.5, respectively. Additionally, the mean value *θ* of the lognormal distribution is set to 2, indicating an average incubation period of 2 days, with a standard deviation *σ* of 1 day. Unless otherwise specified, these assumptions and parameter values remain constant throughout the study. We employ both the MMCA and Monte Carlo (MC) simulations for analysis. The MC simulations are repeated 100 times, and the results are averaged to ensure statistical reliability.

### 3.1 Validation of the MMCA method

The effectiveness of the MMCA method can be validated by comparing its results with experimental data obtained from MC simulations. In the MMCA framework, the steady-state densities of aware nodes and infected nodes are calculated as follows:

(11) $$\rho^{A}=\underset{i}{\sum }\frac{p_{i}^{AS}+p_{i}^{AI}}{N},\;\rho^{I}=\underset{i}{\sum }\frac{p_{i}^{UI}+p_{i}^{AI}}{N}.$$

For the MC simulation, the corresponding values are given by:

(12) $$\rho^{A}=\frac{N^{AS}+N^{AI}}{N},\;\rho^{I}=\frac{N^{UI}+N^{AI}}{N},$$

where *N*^*AS*^, *N*^*UI*^, and *N*^*AI*^ denote the total number of nodes in the *AS*, *UI*, and *AI* states, respectively.

**Fig. 4:**
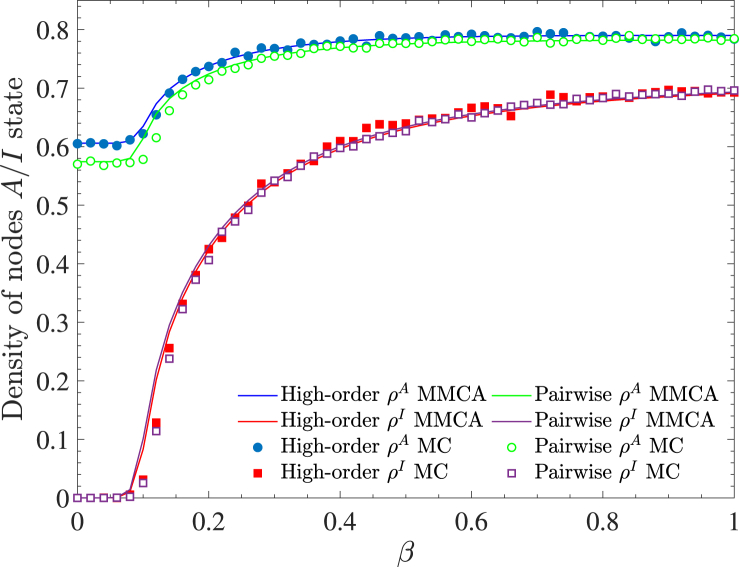
Comparison of *ρ*^*I*^ and *ρ*^*A*^ as functions of *β* between MMCA and MC simulations. The two information propagation modes, high-order and pairwise, are compared. Noting that there is no significant difference of *ρ*^*I*^ between high-order and pairwise, while *ρ*^*A*^ of the high-order is higher than that of the pairwise slightly. The parameter *p*_2_ is set to 0.0008.

Fig. 4 presents the densities of aware and infected nodes obtained through the MMCA method and MC simulations. The close agreement between *ρ*^*A*^ and *ρ*^*I*^ from MMCA (solid line) and MC (symbol) validates the reliability of our model. Notably, the density of aware nodes (*A*-state) is higher than that of infected nodes (*I*-state) at steady state. This is expected, as aware nodes include both aware-susceptible and aware-infected individuals. Another significant observation is that the density of aware nodes, *ρ*^*A*^, is slightly higher under the high-order propagation mode than under the pairwise mode. This confirms that high-order interactions enhance the rate of information dissemination, consistent with findings from Iacopini et al., 2019.

### 3.2 Impact of information propagation on epidemic spread

**Fig. 5:**
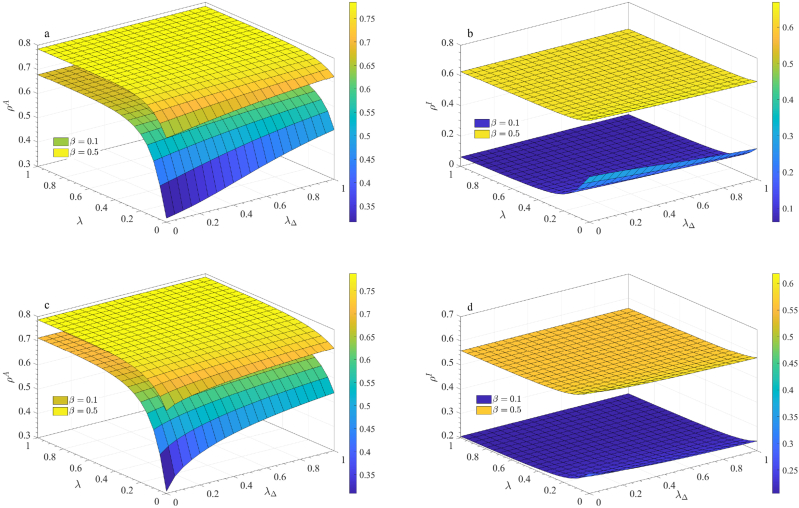
Densities of aware nodes *ρ*^*A*^ and infected nodes *ρ*^*I*^ as functions of the pairwise and 2-simplex information propagation rates (*λ* and *λ*_△_) at steady state. Two infection rates, *β* = 0.1 and *β* = 0.5, are considered. Panels (a) and (b) are obtained from ER-random networks, and panels (c) and (d) are derived from scale-free networks.

The proportions of aware nodes (*ρ*^*A*^) and infected nodes (*ρ*^*I*^) are key metrics for evaluating the model's behavior. Thus, it is crucial to investigate how these proportions vary with different information propagation modes. Fig. 5 shows the variations of *ρ*^*A*^ and *ρ*^*I*^ with respect to *λ* and *λ*_△_ at different infection rates (*β* = 0.1 and *β* = 0.5). Here, *β* = 0.1 represents a relatively low infection rate, while *β* = 0.5 corresponds to a relatively high infection rate, warranting special consideration.

At a low infection rate (*β* = 0.1), when *λ* is small, an increase in *λ*_△_ leads to a rise in *ρ*^*A*^ and a decline in *ρ*^*I*^. This suggests that the reinforcing effect of the 2-simplex not only promotes the spread of disease-related information but also inhibits disease transmission. As *λ* increases, both *ρ*^*A*^ and *ρ*^*I*^ follow the same trend, though with greater magnitude, indicating the dominant role of pairwise interactions. However, at higher infection rates (*β* = 0.5), changes in *ρ*^*A*^ and *ρ*^*I*^ are less pronounced, implying that as more individuals become infected and awareness reaches saturation, the impact of information propagation is significantly reduced. Additionally, we perform the similar experimental operations on scale-free networks, as shown in panels (c) and (d) of Fig. 5, and find that the results are similar to those obtained from ER random networks. It demonstrates consistent trends across different network structures.

### 3.3 Epidemic threshold analysis

Next, we examine the critical condition for an epidemic outbreak, characterized by the epidemic threshold *β*_*c*_, which is an important indicator of disease spread. As previously established, the epidemic threshold *β*_*c*_ is independent of the time delay *τ*_*i*_ and is determined solely by external parameters.

**Fig. 6:**
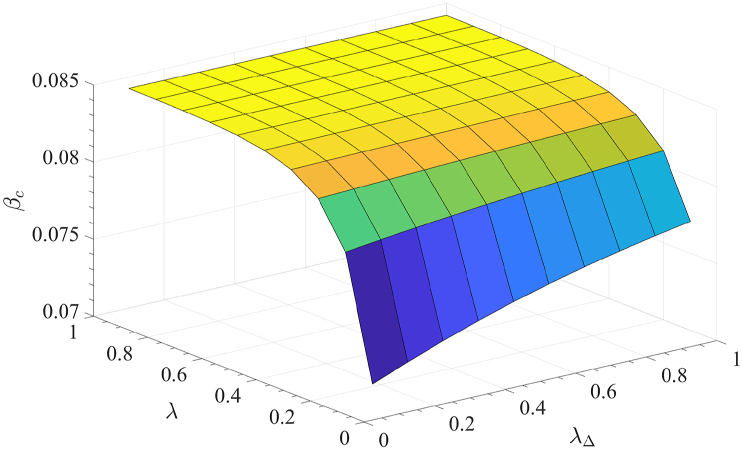
Epidemic threshold *β*_*c*_ as a function of the two information propagation modes (*λ* and *λ*_△_).

To explore how the epidemic threshold is influenced by the two modes of information dissemination, we present the relationship surface in Fig. 6. The results reveal that as *λ* and *λ*_△_ increase, *β*_*c*_ also rises, indicating that a greater spread of awareness elevates the epidemic outbreak threshold. Furthermore, when *λ* is small, an increase in *λ*_△_ results in a noticeable increase in *β*_*c*_. This suggests that awareness propagation via the 2-simplex has a stronger mitigating effect on disease transmission when pairwise interactions are less frequent.

### 3.4 Impact of time-delay

As highlighted in the Introduction, the incubation period of infectious diseases varies among individuals. A key contribution of this study is the development of a coupled disease-information propagation model that incorporates the concept of an incubation period. In this section, we investigate the influence of the disease incubation period (i.e., *τ*_*i*_) on our model.

**Fig. 7:**
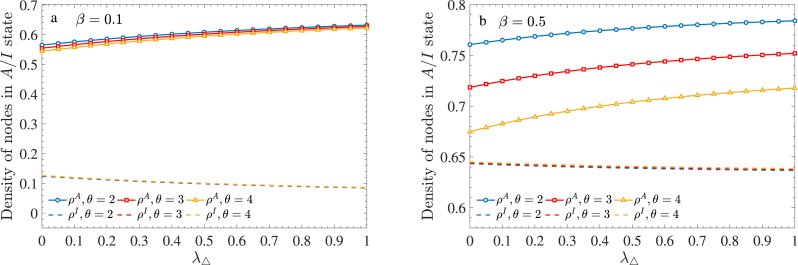
Densities of nodes in the *A*-state and *I*-state as functions of *λ*_△_ for two cases: (a) *β* = 0.1 and (b) *β* = 0.5. Each panel considers three different values of *θ*.

Fig. 7 presents the densities of aware (*ρ*^*A*^) and infected (*ρ*^*I*^) nodes as functions of *λ*_△_ for *β* = 0.1 and *β* = 0.5. In both cases, as *λ*_△_ increases, *ρ*^*A*^ exhibits an upward trend, whereas *ρ*^*I*^ declines slightly. This indicates that the reinforcement effect of 2-simplex interactions facilitates awareness dissemination, thereby mitigating the spread of the epidemic. However, the impact of time-delay on these densities varies with the infection rate. At a low infection rate (*β* = 0.1), the curves representing *ρ*^*A*^ for different values of *θ* nearly overlap, suggesting minimal influence. In contrast, at a high infection rate (*β* = 0.5), the curves are clearly distinguishable, with larger values of *θ* corresponding to lower *ρ*^*A*^. This suggests that an extended incubation period inhibits awareness dissemination more significantly when the infection rate is higher. Notably, regardless of *β*, the curves for *ρ*^*I*^ remain nearly identical, indicating that the incubation period has little effect on disease propagation itself.

**Fig. 8:**
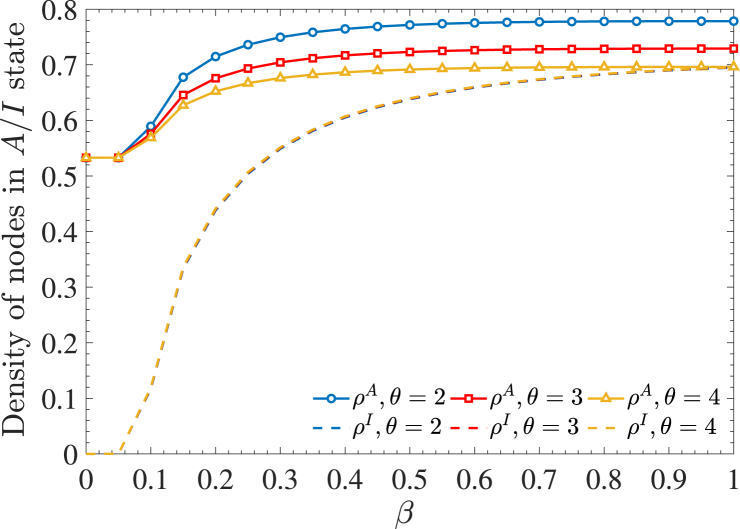
Densities of nodes in the *A*-state and *I*-state as functions of the infection rate *β* for different values of the average incubation period.

To further examine the influence of delayed high-order interactions, we analyze *ρ*^*A*^ and *ρ*^*I*^ as functions of *β* for different values of *θ*, as shown in Fig. 8. As expected, both densities increase with the infection rate. Similar to Fig. 7, the incubation period has no impact on *ρ*^*I*^ but significantly affects *ρ*^*A*^. Specifically, longer incubation periods correspond to lower *ρ*^*A*^, reinforcing the notion that a prolonged incubation period reduces disease-related awareness while leaving disease transmission unchanged.

**Fig. 9:**
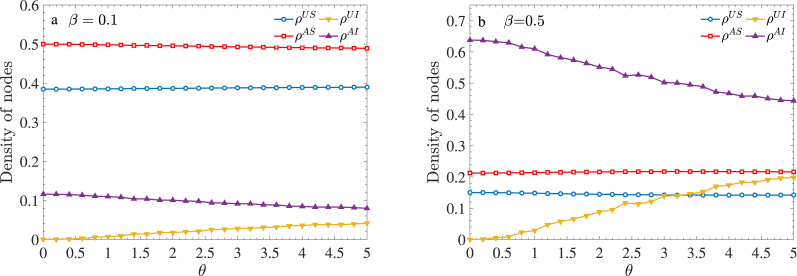
Densities of nodes in the four states of our model as functions of the mean disease incubation period for (a) *β* = 0.1 and (b) *β* = 0.5.

Next, we explore the effect of the incubation period from another perspective by varying *θ* from 0 to 5 days in increments of 0.2 days. We examine the densities of nodes in the four states considered in our model: *ρ*^*US*^, *ρ*^*UI*^, *ρ*^*AS*^, and *ρ*^*AI*^. The results are depicted in Fig. 9.

The results reveal that the primary effect of *θ* is on *ρ*^*UI*^ and *ρ*^*AI*^, whereas the densities of the susceptible states (*ρ*^*US*^ and *ρ*^*AS*^) remain unchanged. This is expected, as the incubation period does not affect the healthy population. As *θ* increases, *ρ*^*UI*^ rises while *ρ*^*AI*^ decreases, irrespective of whether *β* = 0.1 or *β* = 0.5. At a low infection rate (*β* = 0.1), changes in *ρ*^*UI*^ and *ρ*^*AI*^ are minimal. However, at a high infection rate (*β* = 0.5), *ρ*^*AI*^ exhibits significant variation, while *ρ*^*UI*^ experiences a sharp increase. This indicates that a prolonged incubation period leads to a higher proportion of asymptomatic individuals, resulting in a relative decrease in aware individuals. It is important to note that the total density always satisfies the equation *ρ*^*US*^ + *ρ*^*AS*^ + *ρ*^*UI*^ + *ρ*^*AI*^ = 1, implying that susceptible state densities remain at a lower level.

## 4 Discussions

Previous research (Iacopini et al., 2019) has demonstrated that high-order information dissemination amplifies the impact of pairwise transmission on disease spread. Our theoretical analysis and numerical simulations further confirm that, compared to pairwise transmission with time-delay, high-order delayed effects positively influence information propagation related to diseases (See Fig. 4).

We now focus on the independent contributions of the two dissemination methods to information propagation. To quantify this, we use Bayes’ theorem to calculate the conditional probabilities of node *i* being notified via pairwise or 2-simplex propagation. These probabilities, denoted as *P*(*i*^↔^|*i*^*A*^) and *P*(*i*^△^|*i*^*A*^), are given by:

(13) $$\left\{\begin{array}{ll} P\left(i^{↔}|i^{A}\right) & =\frac{q_{1}\left(1-r_{i}^{↔}\right)}{q_{1}\left(1-r_{i}^{↔}\right)+q_{2}\left(1-r_{i}^{△}\right)};\\ P\left(i^{△}|i^{A}\right) & =\frac{q_{2}\left(1-r_{i}^{△}\right)}{q_{1}\left(1-r_{i}^{↔}\right)+q_{2}\left(1-r_{i}^{△}\right)}, \end{array}\right)$$

where *q*_1_ and *q*_2_ represent the proportions of pairwise nodes and 2-simplex nodes in the upper-layer network, satisfying *q*_1_ + *q*_2_ = 1. $r_{i}^{↔}$ and $r_{i}^{△}$ denote the probabilities that individual *i* remains uninformed by aware neighbors through pairwise and 2-simplex propagation, respectively, as given in Eq. (2).

**Fig. 10:**
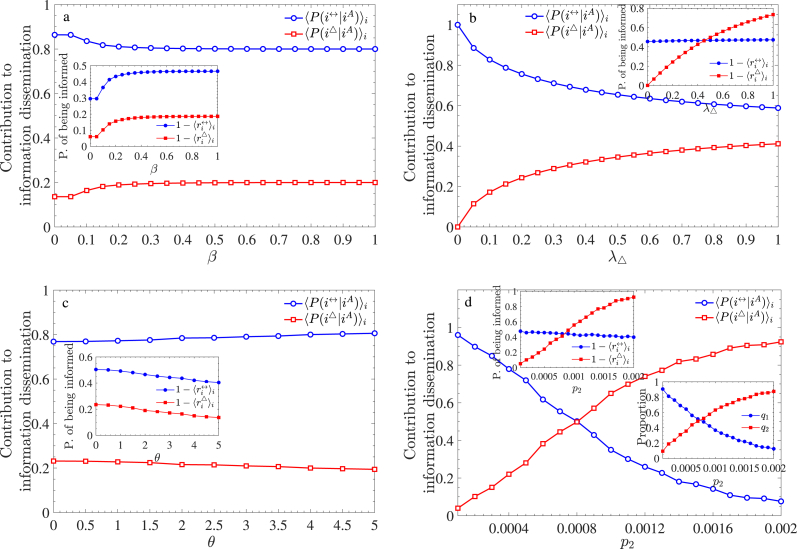
Comparison of awareness dissemination via pairwise and 2-simplex propagation. The main panels show the conditional probabilities as functions of (a) infection rate *β*, (b) high-order information propagation rate *λ*_△_, (c) mean incubation period *θ*, and (d) probability *p*_2_ of forming a 2-simplex in the RSC model. Each inset illustrates the probabilities of an entity being informed through the two propagation modes. The additional inset in panel (d) displays the proportion of pairwise and 2-simplex nodes in the upper-layer network.

We examine the effects of four key parameters—infection rate *β*, high-order information propagation rate *λ*_△_, mean incubation period *θ*, and 2-simplex formation probability *p*_2_—on the two conditional probabilities. The parameter values are consistent with previous settings: *β* = 0.5, *λ*_△_ = 0.15, *θ* = 2, and *p*_2_ = 0.0004. The main panels in Fig. 10 display the average conditional probabilities over all nodes as functions of these factors. In each subplot, one parameter varies while others remain constant. Several key findings emerge:

• As shown in Fig. 10(a) ∼(c), when *β*, *λ*_△_, and *θ* vary, the mean conditional probability ${\left\langle P\left(i^{↔}|i^{A}\right)\right\rangle }_{i}$ remains significantly higher than ${\left\langle P\left(i^{△}|i^{A}\right)\right\rangle }_{i}$. This suggests that pairwise propagation dominates information dissemination due to the sparsity of the network (*p*_2_ = 0.0002). However, as *p*_2_ increases, ${\left\langle P\left(i^{↔}|i^{A}\right)\right\rangle }_{i}$ decreases while ${\left\langle P\left(i^{△}|i^{A}\right)\right\rangle }_{i}$ rises (see Fig. 10(d)).
• In Fig. 10(d), an inset shows that when *p*_2_ ≈ 0.0008, pairwise and 2-simplex nodes exist in roughly equal proportions. For *p*_2_ > 0.0008, 2-simplex nodes outnumber pairwise nodes, making 2-simplex propagation the dominant mode of information dissemination.
• The conditional probabilities show little dependence on *β* and *θ*, implying that disease-related information dissemination is unaffected by the incubation period or infection rate. However, they are somewhat dependent on *λ*_△_ (Fig. 10(b)). As *λ*_△_ increases, the role of 2-simplex propagation expands while that of pairwise propagation weakens. Nonetheless, even with a high *λ*_△_, pairwise propagation remains the dominant dissemination mode due to the small value of *p*_2_.

**Fig. 11:**
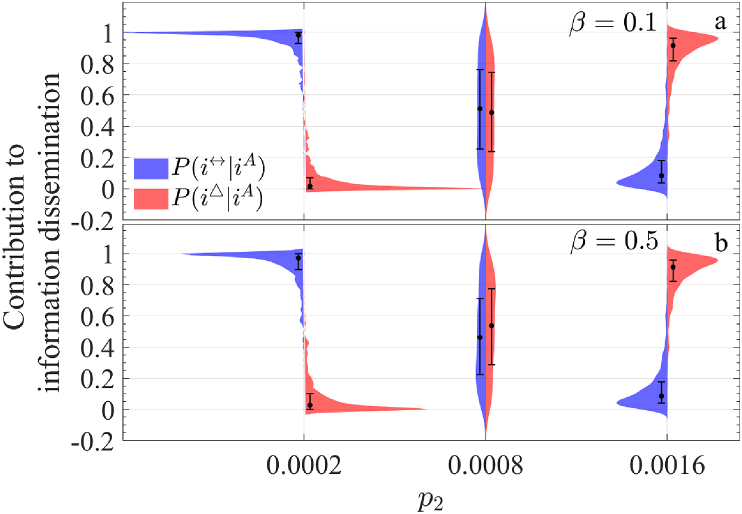
Comparison of the distribution of *P*(*i*^↔^|*i*^*A*^) and *P*(*i*^△^|*i*^*A*^) under three upper-layer network structures (with corresponding to a distinct value of *p*_2_). (a) and (b) represent the case when *β* = 0.1 and 0.5, respectively.

**Fig. 12:**
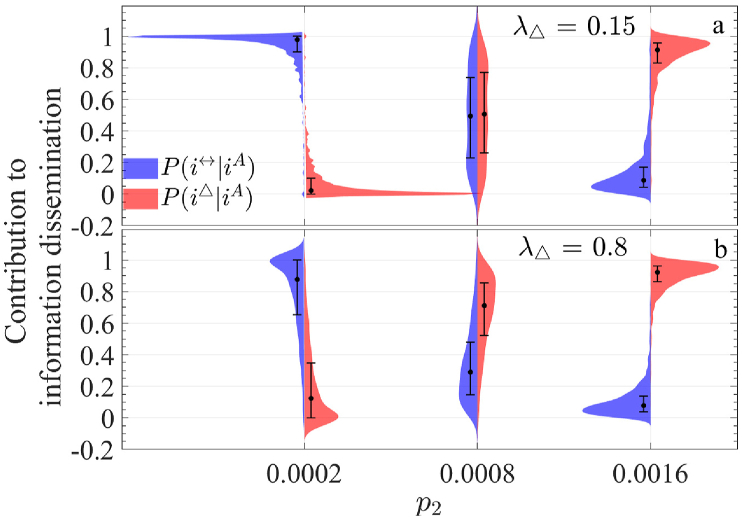
Comparison of the distribution of *P*(*i*^↔^|*i*^*A*^) and *P*(*i*^△^|*i*^*A*^) under three upper-layer network structures (with corresponding to a distinct value of *p*_2_). (a) and (b) represent the case when *λ*_△_ = 0.15 and 0.8, respectively.

**Fig. 13:**
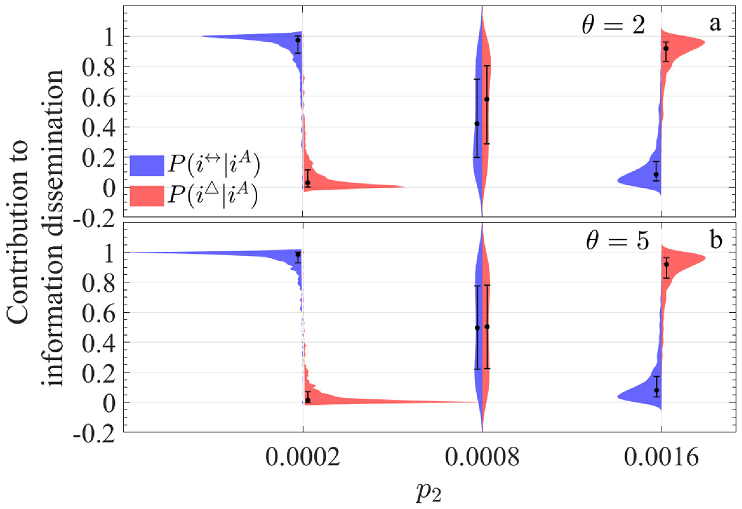
Comparison of the distribution of *P*(*i*^↔^|*i*^*A*^) and *P*(*i*^△^|*i*^*A*^) under three upper-layer network structures (with corresponding to a distinct value of *p*_2_). (a) and (b) represent the case when *θ* = 2 and 5, respectively.

In summary, the contributions of the two modes of information dissemination primarily depend on the network structure, particularly the probability of forming a 2-simplex, *p*_2_. Based on Fig. 10(d), we examine three values of *p*_2_—0.0002, 0.0008, and 0.0016—to compare the relative influence of pairwise propagation and 2-simplex propagation. Fig. 11 ∼13 illustrate the conditional probabilities under varying *p*_2_ for different values of *β*, *λ*_△_, and *θ*, using a network of 1000 nodes. Each figure considers two parameter values: *β* = 0.1 and 0.5 (Fig. 11), *λ*_△_ = 0.15 and 0.8 (Fig. 12), and *θ* = 2 and 5 (Fig. 13).

A clear pattern emerges: when *p*_2_ = 0.0002, *P*(*i*^↔^|*i*^*A*^) exceeds *P*(*i*^△^|*i*^*A*^), whereas for *p*_2_ = 0.0016, the opposite holds. At *p*_2_ = 0.0008, the two probabilities are nearly equal, aligning with Fig. 10(d). However, the distribution shapes differ significantly across these values. The distributions for *p*_2_ = 0.0002 are more concentrated, while those for *p*_2_ = 0.0016 are more dispersed, with maximum variance observed at *p*_2_ = 0.0008.

Additionally, while the distribution patterns for *β* (Fig. 11) and *θ* (Fig. 13) are similar for each respective value, notable differences emerge for *λ*_△_. At *p*_2_ = 0.0002, the distributions are relatively concentrated when *λ*_△_ = 0.15, but become more dispersed at *λ*_△_ = 0.8. This suggests that in sparse networks, higher 2-simplex propagation rates create substantial disparities in information spread across nodes. For *p*_2_ = 0.0008, representing an intermediate 2-simplex scale, the contributions of pairwise and 2-simplex propagation are nearly balanced at *λ*_△_ = 0.15. However, at *λ*_△_ = 0.8, the proportion of pairwise nodes (*q*_1_) significantly exceeds that of 2-simplex nodes (*q*_2_), indicating that 2-simplex propagation has become the dominant mode of information dissemination in this scenario.

## 5 Conclusions

The rapid development of network science has provided powerful tools for analyzing the topological features and dynamic properties of complex systems. This theory has been extensively applied to studying how information propagation influences epidemic spread in complex networks, leading to the development of new models.

In this study, we proposed a novel information-disease coupled propagation model on multiplex networks, incorporating a disease incubation period (time-delay *τ*_*i*_) into a 2-simplex framework. We analyzed how propagation delays affect the steady-state densities of aware and infected nodes. Using a microscopic Markov chain approach, we derived the epidemic threshold, which aligns with Monte Carlo simulation results.

Our extensive simulations demonstrate that the synergistic reinforcement effect of the 2-simplex enhances information spread, increasing public awareness of epidemic situations and strengthening disease prevention efforts. However, the disease's incubation period inhibits information spread, with the inhibitory effect becoming more pronounced as the incubation period lengthens, while having negligible impact on disease transmission. Our model offers a deeper understanding of the intricate interactions between information dissemination and disease spread. These insights provide valuable theoretical support and practical guidance for developing more effective public health policies and interventions.This work establishes a framework for analyzing epidemic spread in multiplex networks; however, several important directions for future refinement remain. One key limitation lies in the assumption regarding node correspondence across layers in the multiplex network. In reality, nodes between layers are not necessarily connected in a one-to-one fashion. The presence of one-to-many correspondences may accelerate the propagation of information across layers, which could, in turn, suppress the spread of diseases to some extent. This aspect is not fully captured in our current model and represents a simplification of real-world interconnected systems. For future work, we plan to incorporate more diverse interlayer connectivity patterns, such as many-to-one and many-to-many relationships, to better understand their influence on epidemic dynamics in coupled multilayer networks. This extension would allow for a more comprehensive analysis of how heterogeneous inter-layer connections shape the overall spreading processes.

## CRediT authorship contribution statement

**Zehui Zhang:** Formal analysis, Methodology, Visualization, and Writing - original draft. **Fang Wang:** Funding acquisition, Supervision, and Writing - review & editing. **Lilin Liu:** Conception and Writing - review & editing. **Lin Wang:** Writing - review & editing.

## Declaration of competing interest

All authors declare that there is no conflict of interest regarding the publication of this paper.
